# In Vivo Low-Temperature Plasma Ionization Mass Spectrometry (LTP-MS) Reveals Regulation of 6-Pentyl-2H-Pyran-2-One (6-PP) as a Physiological Variable during Plant-Fungal Interaction

**DOI:** 10.3390/metabo12121231

**Published:** 2022-12-08

**Authors:** Rosina Torres-Ortega, Héctor Guillén-Alonso, Raúl Alcalde-Vázquez, Enrique Ramírez-Chávez, Jorge Molina-Torres, Robert Winkler

**Affiliations:** 1 Department of Biotechnology and Biochemistry, Center for Research and Advanced Studies (CINVESTAV), Irapuato 36824, Mexico; 2 UGA-Langebio, Center for Research and Advanced Studies (CINVESTAV) Irapuato, Km. 9.6 Libramiento Norte Carr. Irapuato-León, Irapuato 36824, Mexico; 3 Department of Biochemical Engineering, Nacional Technological Institute, Celaya 38010, Mexico

**Keywords:** AIMS LTP-MS, kurtosis, VOCs monitoring, time series in VOCs emissions

## Abstract

Volatile organic compounds (VOCs) comprises a broad class of small molecules (up to ~300 g/mol) produced by biological and non-biological sources. VOCs play a vital role in an organism’s metabolism during its growth, defense, and reproduction. The well-known 6-pentyl-α-pyrone (6-PP) molecule is an example of a major volatile biosynthesized by *Trichoderma atroviride* that modulates the expression of PIN auxin-transport proteins in primary roots of *Arabidopsis thaliana* during their relationship. Their beneficial relation includes lateral root formation, defense induction, and increased plant biomass production. The role of 6-PP has been widely studied due to its relevance in this cross-kingdom relationship. Conventional VOCs measurements are often destructive; samples require further preparation, and the time resolution is low (around hours). Some techniques enable at-line or real-time analyses but are highly selective to defined compounds. Due to these technical constraints, it is difficult to acquire relevant information about the dynamics of VOCs in biological systems. Low-temperature plasma (LTP) ionization allows the analysis of a wide range of VOCs by mass spectrometry (MS). In addition, LTP-MS requires no sample preparation, is solvent-free, and enables the detection of 6-PP faster than conventional analytical methods. Applying static statistical methods such as Principal Component Analysis (PCA) and Discriminant Factorial Analysis (DFA) leads to a loss of information since the biological systems are dynamic. Thus, we applied a time series analysis to find patterns in the signal changes. Our results indicate that the 6-PP signal is constitutively emitted by *T. atroviride* only; the signal shows high skewness and kurtosis. In *A. thaliana* grown alone, no signal corresponding to 6-PP is detected above the white noise level. However, during *T. atroviride-A. thaliana* interaction, the signal performance showed reduced skewness and kurtosis with high autocorrelation. These results suggest that 6-PP is a physiological variable that promotes homeostasis during the plant-fungal relationship. Although the molecular mechanism of this cross-kingdom control is still unknown, our study indicates that 6-PP has to be regulated by *A. thaliana* during their interaction.

## 1. Introduction

Pathogens and diseases cause around 20 to 40% of agricultural losses worldwide [1]. Biocontrol agents are an alternative to agrochemicals to avoid pests, improve plant growth, and produce sustainable production yield. For instance, *Trichoderma* species are widely used. The biological functions of this fungus include mycoparasitism, competition with other fungi for space and nutrients, antibiosis, stimulation of lateral root development, degradation of toxic compounds, enhancing nutrient solubility, mineral acquisitions, and induction of plant defense response [2]. The beneficial activity of *Trichoderma* depends on their ability to establish molecular signaling with the plant, actively secreting and exchanging molecules such as cell wall degrading enzymes, siderophores, volatile and non-volatile metabolites [3].

Volatile Organic Compounds (VOCs) are gases-phase and carbon-based molecules characterized by low molecular weight and high vapor pressure. All living beings produce VOCs from their specialized metabolism, and their particular production responds to internal and external stimuli. The chemical diversity of VOCs includes alcohols, ketones, esters, alkenes, monoterpenes, sesquiterpenes, among others [4,5,6,7].

Volatiles play essential roles as signaling molecules during interactions among organisms. For example, some volatiles of fungal origin (fVOCs) have important effects on plant growth and defense [2,8]. The 6-Pentyl-α-Pyrone (6-PP) is a very common fungal-produced volatile with the popular “coconut” aroma [3]. Adding 6-PP to plant growth media or directly applying it to their leaves increases root number and biomass production [9,10]. The 6-PP can also inhibit the growth of several plant pathogens, such as *Botrytis cinerea*, *Phytophthora megasperma*, *Rhizoctonia solani*, *Armillaria mellea*, and *Fusarium* spp. [3].

Moreover, this fVOC participates in multiple plant defense responses such as Reactive Oxygen Species (ROS) production; anthocyanins, phytoalexin, and camalexin accumulation; increasing the number of trichomes; activate defense genes, mainly associated with salicylic acid (SA) pathway; and increased glucosinolates levels in plants [10,11]. However, it is still unknown how this molecule interacts with the plant and how it is regulated.

The principal method for the study of volatiles is gas chromatography (GC), coupled with flame ionization detection (FID) or mass spectrometry (MS). Usually, the VOCs are collected first on solid-phase microextraction (SPME) fibers. GC methods require considerable sample preparation and have limited time resolution [2]. A complete SPME-GC-MS takes at least an hour. In addition, the SPME fibers only trap part of the VOCs, depending on the chosen material, and could become saturated by abundant VOCs, masking other VOCs that might be biologically relevant.

In stark contrast, new ambient ionization MS (AIMS) methods enable life-compatible real-time measurements [12,13,14]. Introduced in the early 2000s, ambient ionization mass spectrometry (AIMS) represents a revolution in chemical analysis, and complex mixtures can be analyzed without previous sample manipulation [15]. In addition, these methods are suitable for in vivo and in situ studies [16]. The first ambient ionization MS described was the solvent-based desorption electrospray ionization (DESI) technique [17], closely followed by plasma-based direct analysis in real-time (DART) [18]. Rapidly, numerous ambient techniques have been reported [19,20]. Furthermore, improvements in the design and analytical performance of different methods helped in their application in various fields, from forensics to clinical analysis, including imaging [21,22,23].

One of these ambient ionization methods is LTP coupled with MS, first reported by Harper et al. [24], which has many advantages over other ionization sources:

1. LTP allows chemically very diverse molecules to be ionized, since the plasma simultaneously contains positively or negatively charged particles.
2. LTP systems are adaptable to any MS with an accessible atmospheric inlet, e.g., coupling with linear ion trap, quadrupole, or Fourier Transform-Ion Cyclotron Resonance (FTICR) spectrometers.
3. The operating conditions of LTP-MS systems are more straightforward than conventional mass spectrometry ionization sources such as EI, ESI, or MALDI.
4. LTP is solvent-free, and the temperature is kept below 40 °C. [16,23].

LTP-MS has previously been used to study plant metabolite production, quality control of market products, pharmaceutical, petrochemical, microorganism identification, and imaging of volatile and semi-volatile compounds effectively [14].

External and internal stimuli can modify an organism’s particular State of Health (SoH), a parameter that can be assessed by measuring the metabolic activity of organisms [25]. Therefore, VOCs can reflect SoH, and biological processes can be monitored through volatile emissions; for example, some human diseases are detectable by measuring these molecules [26]. Compensatory mechanisms, namely homeostasis, control SoH. Homeostasis is a term that joins two key ideas: (i) the internal *stability* of an organism and (ii) coordinated adaptive responses to maintain this internal stability [27]. Claude Bernard first defined ‘*homeostasis*’ as the ability to keep the internal environment constant [28,29,30]. Monitoring homeostasis in living systems is not trivial. We usually take punctual measurements of biological parameters (for example, temperature, blood pressure, and pH, etc.). However, continuous monitoring of physiological variables is still challenging due to technological limitations and the lack of data analysis tools [27,29]. The introduction of time series analysis to calculate homeostasis is a novel topic. For example, Fossion et al. [27] reported using different algorithms based on time series analysis to study variables of diabetes and non-diabetes patients, such as blood pressure and heart rate. However, VOC production analysis using time-series algorithms has not been reported until now. In the Fossion et al. study, heart rate was identified as a physiological variable, since, contrary to other types of variables called regulated (such as blood pressure), the heart rate in healthy patients was characterized by its high variability and adaptability. On the contrary, in patients with diabetes, this variability decreased, losing the adaptability era of the system. Thus, they were able to demonstrate how the behavior of these variables contributes to the health of the organism.

In this work, we use a low-temperature plasma (LTP) ionization source coupled with mass spectrometry (MS) to measure the dynamics of 6-PP production by *T. atroviride* during interaction with *A. thaliana*. The 6-PP production pattern analysis showed this molecule as a physiological variable that follows the rules of homeostasis in an ecological two-organism system. Furthermore, we show that time series analysis of AIMS data enables studying the VOC’s pattern dynamics in biological systems.

## 2. Methods

### 2.1. Biological Material

**Figure 1:**
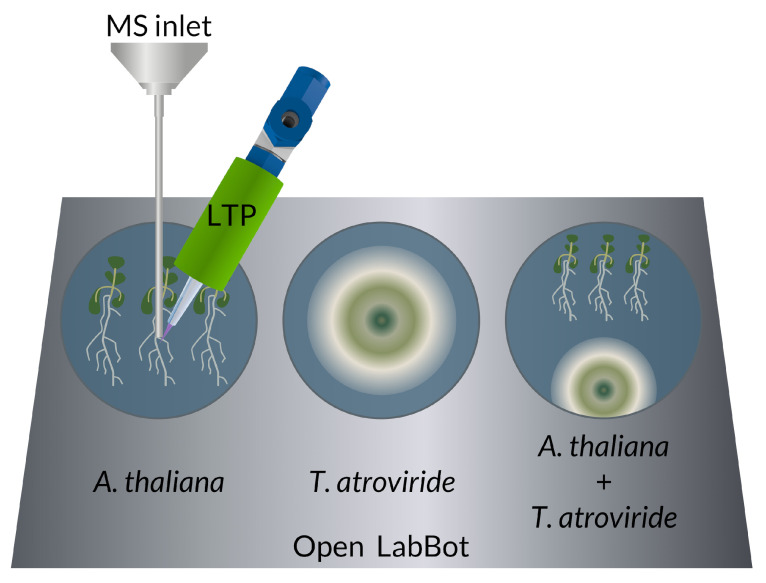
Experimental set-up for studying the plant-fungal interaction between *Arabidopsis thaliana* and *Trichoderma atroviride* in vivo with low-temperature (LTP) ionization mass spectrometry (MS).

*Arabidopsis thaliana* Col-0 seeds were disinfected with 70% (*v*/*v*) ethanol for 1 min, washed with chlorine 20% (*v*/*v*), and rested for seven minutes. Next, the seeds were washed with deionized sterile water four times. Finally, seeds were suspended in 300 μL of sterile water and kept at 4 °C for three days in darkness (Figure 1).

*Trichoderma atroviride* was grown on Luria–Bertani (LB) solid media, and spores were collected on 50% of glycerol *v*/*v*. Spores were counted on a Neubauer chamber and stored at −80 °C until their use.

### 2.2. Plant Growth Conditions

We planted the *A. thaliana* seeds in plates with 0.2× of Murashige & Skoog medium (MS) (Sigma), 30 g/L of saccharose (Sigma), 7 g/L of agar, and a pH adjusted to 5.7 with hydrochloric acid (HCl) (Sigma) solutions. In each plate, 20 seeds were grown in a photoperiod of 16:8 h of light: dark with a temperature of 22 °C for five days. Plates were placed at 65° relative to the horizon.

### 2.3. Infection with T. atroviride

After five days of growth, *A. thaliana*, was inoculated with 8 × 10^4^ spores of *T. atroviride*. Plates were analyzed immediately at room conditions. Plates were placed at 65° relative to the horizon.

### 2.4. 6-PP Determination by Standardized SPME-GC-MS

To identify the 6-PP molecule using a standard method, we performed a Solid-Phase Microextraction (SPME) on days 3 and 5. The fiber (fused silica fiber plain blue hub for volatiles) was exposed for 1 h. The injection sample port was at 230 °C to introduce the compounds in an Agilent 19091S-433 HP-5 ms column. GC-MS detected VOCs in the following conditions: start at 45 °C with a gradient of 9 °C/min up to 250 °C, maintaining the temperature for 1 min, and decreasing it to 220 °C, as described in [11]. The data were compared to the National Institute of Standards and Technology (NIST) 2008 database, with Enhanced ChemStation (v. F.01.01.2317) from Agilent Technologies with a spectra similarity of 87.4% [31].

### 2.5. 6-PP Determination by LTP-MS

For ambient ionization mass spectrometry (AIMS), we used a 3D-printed low-temperature plasma (LTP) probe, described in [32].

Aliquots of 6-PP standard (Sigma) were exposed directly to the LTP-MS system. The molecular ion was detected at 167.1 [M + H]^+^ *m/z* and fragmented at 30 eV on an LCQ Fleet ion trap (Thermo Scientific, USA).

The identity of 6-PP in the *T. atroviride-A. thaliana* monitoring experiments were confirmed on the third and fifth days by direct LTP-MS. The 167.1 *m/z* ions were detected, isolated, and fragmented at 30 eV and compared with the genuine standard (Sigma, Mexico).

**Figure 2:**
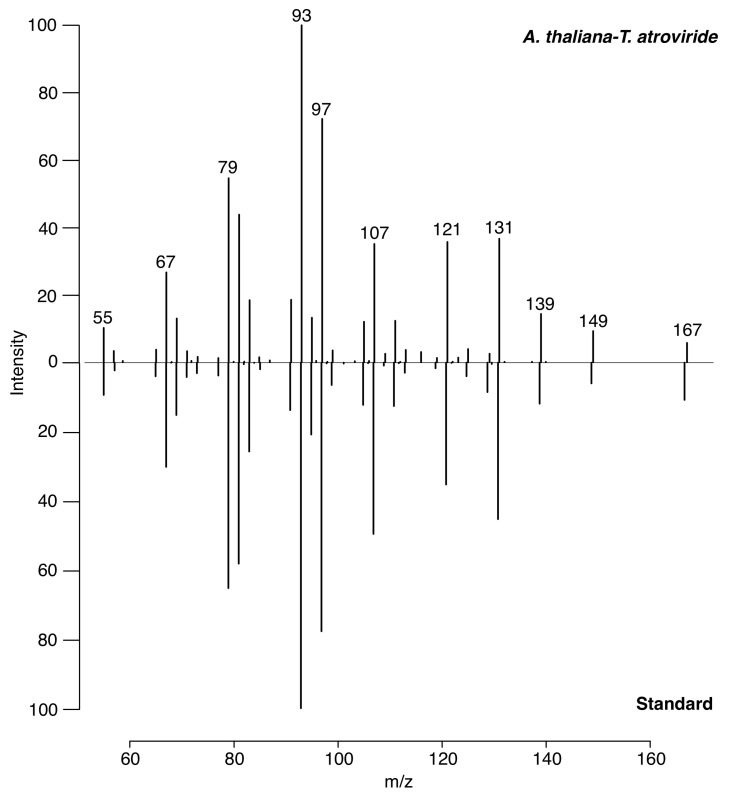
Fragmentation of 6-PP, measured by low-temperature plasma (LTP) ionization mass spectrometry (MS), on an ion trap with 30 eV collision energy. The spectra from the plant-fungal interaction (top) and a genuine 6-PP standard (bottom) demonstrate high agreement, confirming the identity of 6-PP.

A comparison between an experiment’s 30 eV fragmentation spectrum and the genuine standard is demonstrated in Figure 2.

### 2.6. 6-PP Kinetics and Other VOCs by LTP-MS during T. atroviride-A. thaliana Interaction

Full MS spectra were taken from medium plates, *A. thaliana*, *T. atroviride,* and co-cultivation every hour, starting with the inoculation. For automated measurements, we used the open LabBot [33]. We cleaned the MS inlet with methanol for 1 min before each measurement. We tested each condition with three biological replicas and three technical replicas composed of 10 micro scans per minute.

The kinetics of 6-PP were analyzed by a mean of the three biological samples and obtained its standard deviation at each measurement. To quantify 6-PP production, the area under the curve of the mean of biological replicates were obtained.

### 2.7. Data Analyses

We used the Julia programming language (https://julialang.org/), version 1.5.4, for this study. Kurtosis, autocorrelation, and skewness were obtained using the ‘StatsBase’ package. For standard plots, we used the ‘Plots’ package. We developed the function for the Poincaré chart. The complete time-series analysis pipeline is available from (https://github.com/CINVESTAV-LABI/mobims_analysis).

## 3. Results

### 3.1. Identification of 6-PP by SPME-GC-MS

**Figure 3:**
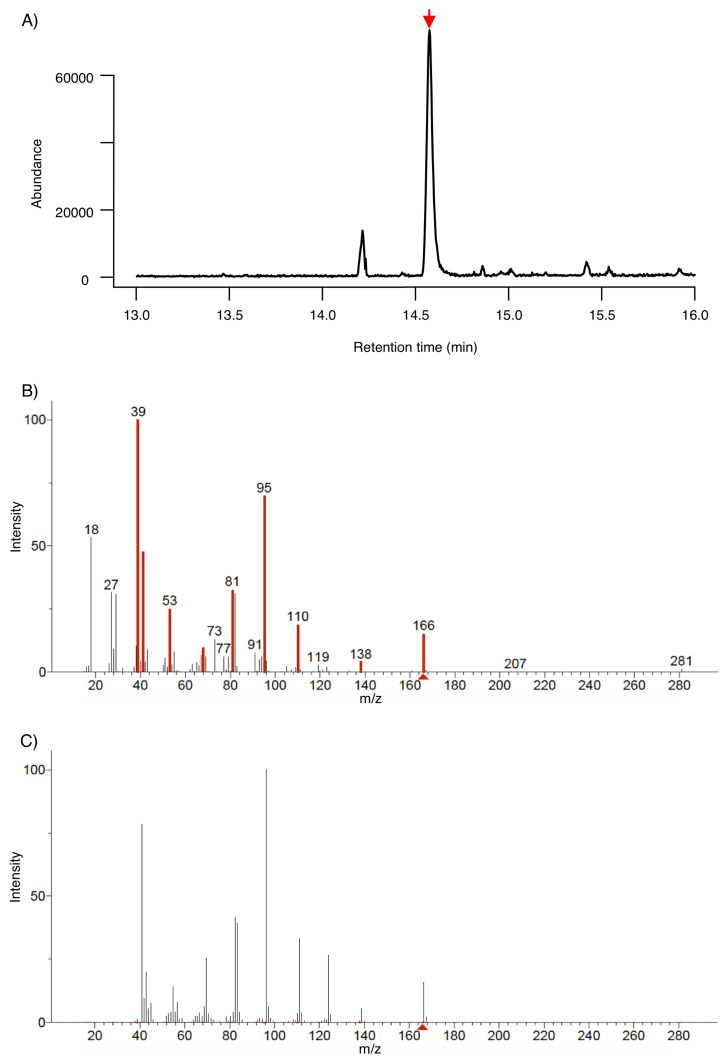
Identification of 6-pentyl-α-pyrone (6-PP) using gas chromatography coupled with mass spectrometry (GC-MS). (**A**) Chromatogram based on Total ion current (TIC), 6-PP was detected at minute 14.6. (**B**) Experimental GC-MS spectrum at 14.6 min. Fragments that match the 6-PP standard fragmentation pattern are highlighted in red. (**C**) NIST database hit of 6-PP.

After three days of *A. thaliana*-*T. atroviride* interaction; we identified 6-pentyl-α-pyrone (6-PP) using SPME-GC-MS. A peak at approximately 14 min indicated the presence of the compound of interest. We identified 6-PP by matching the experimental fragmentation pattern with the NIST database (Figure 3A–C).

### 3.2. Identification of 6-PP by LTP-MS

The molecular ion of 6-PP was detected at 167 *m/z* [M + H]^+^ with LTP-MS. We confirmed its identity by comparing the fragmentation pattern of this ion with a genuine standard compound (Figure 2).

### 3.3. Time Series of 6-PP Kinetics Production

#### 3.3.1. Time Evolution and Behavior

**Figure 4:**
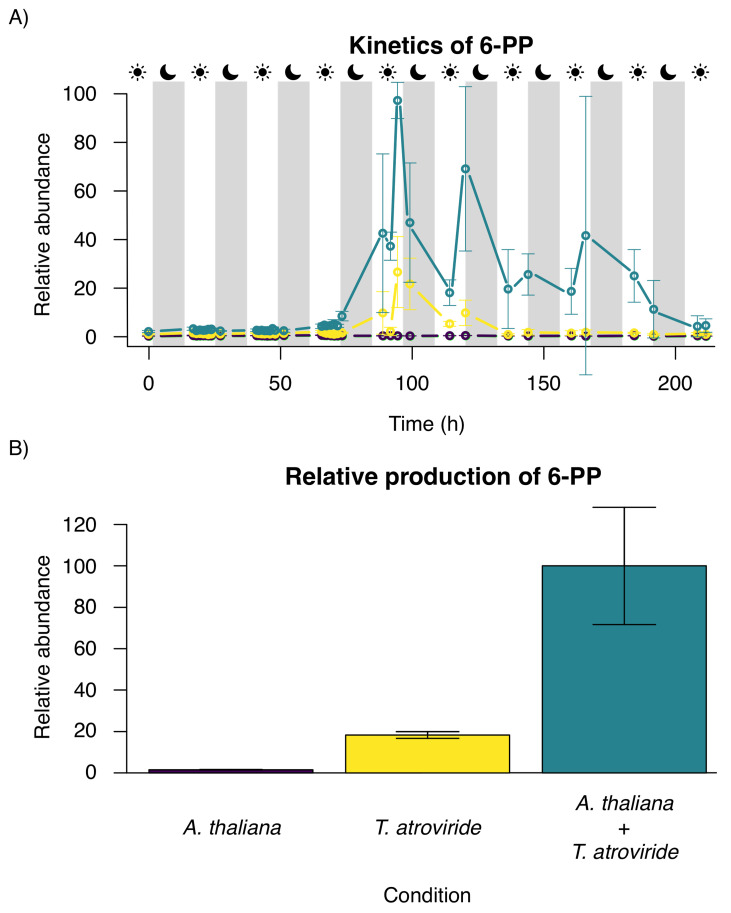
Monitoring of 6-PP production with low-temperature plasma (LTP) ionization mass spectrometry (MS). The mean value and the standard deviation for each measurement are shown. (**A**) The observed 6-PP concentration at night is higher, suggesting a circadian regulation. (**B**) The plant-fungal interaction between *Arabidopsis thaliana* and *Trichoderma atroviride* shows much higher levels of 6-PP than the individual pure cultures. The relative 6-PP production corresponds to the sum of all measurements.

The time course experiment, shown in Figure 4A, indicates that the 6-PP signal is consecutively emitted by *T. atroviride* only; the maximum amount of 6-PP production was achieved on the fifth day when *A. thaliana* and *T. atroviride* were co-cultivated (Figure 4A). The production of 6-PP during the night is higher than during the day, indicating a circadian regulation in *T. atroviride*. The 6-PP overall production of the consortium was about five times increased compared to the pure culture of *T. atroviride* and also lasted two days longer (Figure 4B).

#### 3.3.2. Frequencies and Statistical Moments

**Figure 5:**
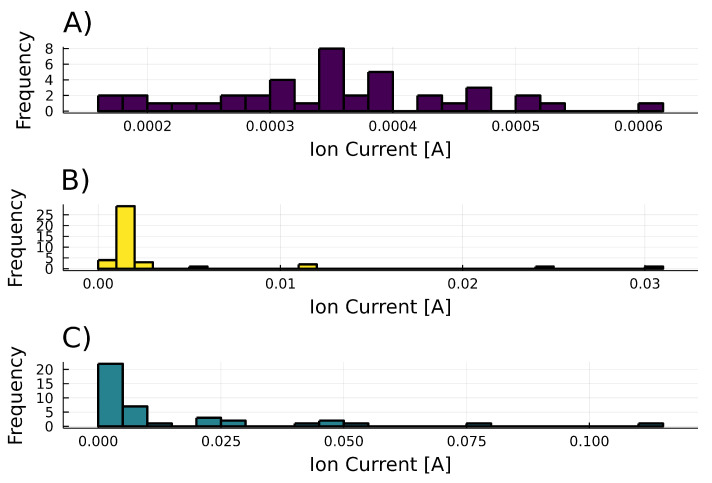
Histograms of the 6-PP ion signal during the experimental time course. (**A**) *Arabidopsis thaliana* (background, no 6-PP production detected) (**B**) *Trichoderma atroviride* (constitutive production of 6-PP at low level). (**C**) Interaction between *Arabidopsis thaliana* and *Trichoderma atroviride*.

In Figure 5A, at the frequency domain, *A. thaliana* 6-PP production exhibits a Gaussian-like behavior, similar to white noise, representing the plant’s lack of the 6-PP production. In contrast, Figure 5B, 6-PP production in *T. atroviride* alone shows high asymmetry; physiological variables in human health have demonstrated this characteristic. In Figure 5C, when both organisms interact, frequency is balanced in between, exhibiting an asymmetrical pattern, but less than in *T. atroviride* alone. In Table 1, we summarize statistical moments. *A. thaliana* signal emission is near zero, while *T. atroviride* signal emission illustrates high kurtosis and skewness. When the two organisms grow together, both statistical moments decrease and stabilize.

#### 3.3.3. Autocorrelations

**Figure 6:**
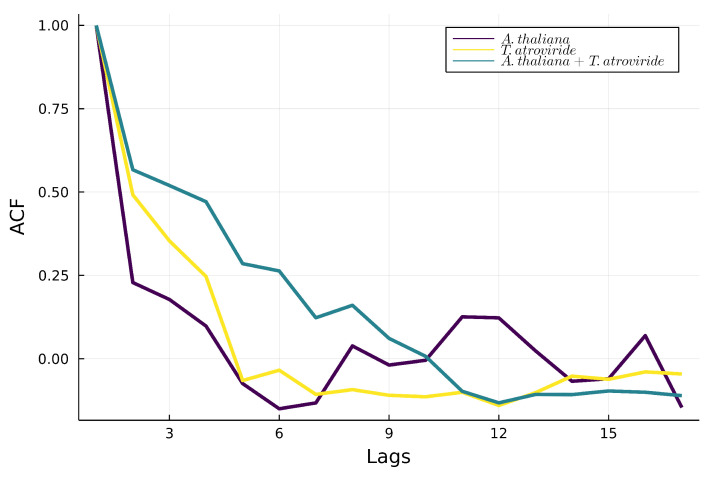
Autocorrelation of the 6-PP signal for the individual organisms: *Arabidopsis thaliana* (purple line), *Trichoderma atroviride* (yellow line), and (C) *A. thaliana + T. atroviride* interaction (green line).

In Figure 6A, the 6-PP signal in *A. thaliana* shows low autocorrelation, characterized by random behavior. In contrast, the signal in the culture of *T. atroviride* reflects higher autocorrelation than the culture of *A. thaliana* (Figure 6B; however, in Figure 6C, the autocorrelation exhibited during the interaction between *A. thaliana* and *T. atroviride* behaves as the sum of the two individual correlations.

#### 3.3.4. Poincaré Plot

**Figure 7:**
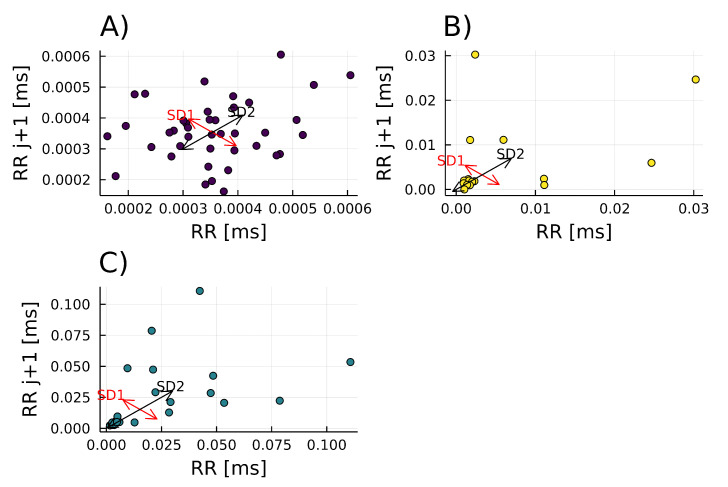
Poincaré of the 6-PP signal for the individual organisms (**A**) *Arabidopsis thaliana* (purple dots) (SD1 = 8.6795 $\times \;10^{-5}$, SD2 = 0.0001, SD1/SD2 = 0.7789), (**B**) *Trichoderma atroviride* (yellow dots) (SD1 = 0.004, SD2 = 0.007, SD1/SD2 = 0.58816), and (**C**) *A. thaliana - T. atroviride* interaction (green dots) (SD1 = 0.01545, SD2 = 0.0295, SD1/SD2 = 0.5222).

The *A. thaliana* signal reflects a low correlation of one point to the next; its behavior is highly random (SD1/SD2 = 0.7789), and it depends only on the equipment measurements since *A. thaliana* does not produce 6-PP, as seen in Figure 7A. In contrast, the *T. atroviride* Poincaré plot has an elliptical shape, which means previous points do not determine the next ones (SD/SD2 = 0.58816); its behavior is somewhat random (Figure 7B). For the interaction of *A. thaliana* with *T. atroviride*, the Poincaré plot shows an intermediate (SD1/SD2 = 0.5222) of both previous conditions (Figure 7C).

## 4. Discussion

*Homeostasis* is a fuzzy concept that has been defined multiple times. Originally, homeostasis was described as the main property of individual organisms, where a set point or ‘balance’ has to be maintained for an organism to function correctly; the balance is achieved through special internal regulation such as control theory found in engineering systems [28,34,35]. However, this concept has been extended by studying homeostasis at broader biotic levels.

From an ecological point of view, homeostasis is the tendency of an ecosystem to maintain the stability of specific properties, such as energy, productivity, biomass, or nutrient flux, despite abiotic environmental perturbations or changes in the biotic composition [36,37]. Moreover, homeostasis has been described as a property that can emerge in a system with diverse biological components and multiple environmental variables.

On a macro level, the ‘*Gaia hypothesis*’ states that the earth, with its biota, acts as a homeostatic geophysiological system that regulates global properties [38,39]. In this work, we study the role of a volatile compound in the homeostatic balance between two species.

Two main paradigms have quantified homeostasis. The first one is the early-warning signals, in which a set point has to be maintained to perform correctly. This regulation is assured by different control systems or feedback loops within individuals. On the contrary, the critical transitions paradigm explains that physiological variables have to exhibit high variability and non-Gaussian distribution increases in adverse conditions.

New studies have bridged both views in a single one where homeostasis balances robustness and adaptability. In this view, variables are regulated, non-regulated, or physiological. Regulated variables must be tightly controlled, while physiological variables respond to external stimuli and exhibit non-Gaussian behaviors [27].

### 4.1. Dynamics of 6-PP Production in Plant-Fungal Interaction

In this study, we calculated statistical moments and time series algorithms to show that 6-PP is a physiological variable in the interaction of *Arabidopsis thaliana* and *Trichoderma atroviride*.

6-PP modulates the transporters of the auxin signaling pathway, inducing the formation of lateral roots (TIR, AFB2, and AFB3 receptors and ARF7 and ARF19 transcription factors). In addition, EIN2, a key element in the ethylene signaling pathway, is also involved in detecting the 6-PP molecule in primary roots [11].

To understand the possible role of 6-PP as a physiological variable, we monitored 6-PP production by *T. atroviride* as part of the volatiles secreted by fungal colonies alone or in interaction with *A. thaliana* plants. LTP-MS analysis revealed that the production of 6-PP is higher in the presence of *A. thaliana*. During the interaction, 6-PP production was detected from the third to the eighth day, and the maximum production was reached on day 5th, which correlates with previous studies [3,5]. Although VOCs secreted by *T. atroviride* include hundreds of compounds, 6-PP has been identified as the most prominent VOC in some *Trichoderma* spp.

We also found that 6-PP production increases when the night begins. We measured the highest concentration of 6-PP at 20:00 (Figure 4). A recent study showed that the circadian clock of T. atroviride regulates the 6-PP molecule and that 6-PP production is highest upon dark incubation, which agrees with our results [40,41]. Moreover, another study demonstrated an increase in 6-PP at night and a decrease in other VOC types [42].

The emission of 6-PP by *T. atroviride* alone is present and exhibits high skewness and kurtosis. As a result, its production shows a non-Gaussian distribution, a significant characteristic of physiological variables (Table 1). On the other hand, when interaction *A. thaliana* - *T. atroviride* exists, the production of 6-PP increments but the skewness and kurtosis decrease, which expresses the system’s stability when both organisms are present. Moreover, 6-PP production when *A. thaliana* - *T. atroviride* interact exhibits high autocorrelation (Figure 6). The autocorrelation parameter measures the relationship between a variable’s current and past values. In other words, 6-PP demonstrates modulation during the interaction, for its production is separated by various time lags.

In contrast, the autocorrelation of 6-PP by *T. atroviride* alone is lower. Biological variables are classified into regulated and physiological variables within a physiology framework. Regulated variables remain constant and show normal statistics, physiological variables adapt to disturbance to ensure the system’s stability, and normal behavior is not presented [43,44]. Thus, we designate the 6-PP as a physiological variable.

Another important recurrence plot is the Poincaré diagram, which is usually a geometrical and nonlinear method to assess heart rate variability (HRV) dynamics [27]. However, it also expresses self-similarity in processes. In this case, when *A. thaliana* and *T. atroviride* are interacting. The Poincaré plot describes an intermediate response (SD1/SD2 = 0.5222), not too deterministic nor too random, suggesting that 6-PP is under homeostatic regulation (Figure 7C). On the contrary, when *T. atroviride* alone, 6-PP emission indicates higher randomness (SD1/SD2 = 0.58816). The system represents an example of two organisms utilizing VOCs as trans-kingdom signals to establish a relationship.

Most studies are focused on the *T. atroviride* production of metabolites and how this organism affects *A. thaliana* growth and defense responses. However, little is known about how *A. thaliana* could modulate this beneficial relationship as feedback control. 6-PP is probably synthesized from linoleic acid using reduction, β-oxidation, and isomerization processes [2]. However, the exact biosynthesized pathway for the production of 6-PP remains to be elucidated, and a lipooxygenase gene unique to *T. atroviride* may be involved. A mutation in the G alpha subunit gene TG1 of *T. atroviride* diminishes 6-PP production and elevates internal cAMP concentrations and continuous sporulation. Furthermore, it is documented that the transcriptor factor Thctf1 is involved in 6-PP production and antifungal activity of *T. harzianum* [25].

### 4.2. Time-SERIES Analysis of Biological MS Data with Julia

Apart from the technical challenges, data analysis is also defiance. Analyzing the data with complex systems and a time series approach allows for finding relevant parameters for the physiology of living systems. The time evolution or time series of these variables is conjectured to reflect the dynamics of the underlying homeostatic regulatory mechanisms and to correlate with the health status of the organ, process, or system under study [27,45]. Time series in *Trichoderma* alone have been studied to investigate the chemical diversity of four *Trichoderma* spp., using two different mass spectrometry techniques. Guo et al. [46] developed a platform for VOC-based chemotyping for a real-time headspace analysis based on multivariate data analysis and machine learning to obtain volatile metabolic fingerprints.

Combining bio-compatible technology and data analysis with complex systems and a time series approach, we managed to track 6-PP and observe that it acts as a physiological variable during *A. thaliana* - *T. atroviride* interaction.

For the statistical analyses, we used the open-source programming language Julia https://julialang.org/ and published the code of our study (see Section 2). Julia programs demonstrate high execution speeds, unlike other programming languages commonly used for MS data analysis, such as R, Python, and Java [47]. Thus, Julia is suitable for the time-critical processing of biological MS data.

### 4.3. LTP-MS for Monitoring VOCs in Biological Systems

VOC monitoring can indicate some organisms’ State of Health (SoH). However, VOC studies have been hampered by the need for proper methods, techniques, and dynamic assessments. VOC identification is usually achieved by gas chromatography (GC) coupled to FID and MS analyzers. Proton-transfer reaction (PTR) MS [48] is suitable for ‘in situ’ analysis but is costly for routine studies and cannot detect all compounds. Structure characterization and identity confirmation are achieved by matching mass spectra and linear retention induced using GC-MS solution with NIST libraries. 6-PP is usually detected by TLC and HPLC analyses based on ethyl acetate extraction. However, various sample processing steps are necessary for obtaining the crude extracts (filtration, harvesting, drying and evaporating, solubilizing, and analyzing); therefore, the original dynamics are lost [2].

The Low-Temperature Plasma coupled to Mass Spectrometry (LTP-MS) [32]; is suitable for in vivo monitoring of VOCs under ambient conditions, allowing follow biological processes through their volatile emissions. In this study, we show that LTP-MS is also a suitable method for 6-PP monitoring.

Current methods for VOC measurements require highly skilled human resources. We compared LTP-MS with SPME-GC-MS, and the following problems were identified in the SPME-GC-MS technique:

1. High fiber saturation with water results in poor VOC capture.
2. Molecules are lost because of fiber selectivity.
3. Extensive time of capture and separation for molecule identification, 1.3 h at least between both phases.

For this study, LTP was superior to SPME-GC-MS because time and sample preparation steps were reduced, and higher time resolution was obtained.

The ambient conditions of the LTP-MS measurements enabled the direct study of VOC production during the plant-fungus interaction without any sample manipulation. Thus, LTP-MS facilitates the collection of biologically meaningful data.

Coupling LTP ionization to miniature mass analyzers such as the Mini 12 [49] and the MoBiMS [12] will further support the development of biocompatible MS technologies.

## 5. Conclusions

We studied the homeostasis of a plant-fungal interaction by combining ambient low-temperature plasma (LTP) ionization mass spectrometry (MS) with time series analysis.

*Arabidopsis thaliana* modulated the production of 6-pentyl-α-pyrone (6-PP) of the fungus *Trichoderma atroviride*. Since *A. thaliana* is not capable of 6-PP biosynthesis when grown alone, the plant must regulate the 6-PP level as a physiological variable during the interaction.

Our results demonstrate the tremendous potential of studying volatile organic compounds (VOCs) in biological systems with in vivo ambient mass spectrometry methods such as low-temperature plasma (LTP) ionization.

## Figures and Tables

**Table 1:** Time series model of *Arabidopsis thaliana* interactions with *Trichoderma atroviride*.

Condition	Skewness	Kurtosis
*A. thaliana*	0.424891559	−0.319935009
*T. atroviride*	3.51034121	11.65552257
*A. thaliana + T. atroviride*	2.442766689	6.019413496

## Data Availability

Not applicable.

## Abbreviations

We followed the “Periodic Table of Mass Spectrometry (MS) Terms” [50]. The following abbreviations are used in this manuscript:

6-PP	6-Pentyl-2H-pyran-2-one
AIMS	ambient ionisation mass spectrometry
DART	direct analysis in real-time
DESI	desorption electrospray ionisation
EI	electron impact
ESI	electrospray
FID	flame ionization detection
fVOC	fungal volatile organic compound
GC	gas chromatography
HCl	hydrochloric acid
HPLC	high-performance liquid chromatography
HRV	heart rate variability
LTP	low-temperature plasma
LB	Luria-Bertani
MALDI	matrix-assisted laser desorption/ionisation
MA	methamphetamine
MS	mass spectrometry
MS medium	Murashige & Skoog medium
NIST	National Institute of Standards and Technology
PTR	proton-transfer reaction
ROS	reactive oxygen species
SA	salicylic acid
SoH	state of health
SPME	solid-phase microextraction
TIC	total ion current
VOC	volatile organic compound
